# Walter Salisbury

**Published:** 1935

**Authors:** 


### Obituary

WALTER SALISBURY, M.D., M.S., F.R.C.S.

MR. WALTER SALISBURY died suddenly at Northampton on 7th January, 1935, at the age of forty-six. He had been suffering from pneumonia following influenza. He entered the Bristol Medical School in 1906, and was a student at the Royal Infirmary, winning the Tibbits Prize for Surgery. He qualified M.B., B.S. London, in 1913, and held the offices of House Surgeon and Casualty Officer at the Royal Infirmary and Senior Resident Officer at the General Hospital; he also held resident appointments at Queen Charlotte's Hospital, at the Hospital for Women, Soho, and at the Royal National Orthopædic Hospital. During the War he obtained a commission in the R.A.M.C., and served as a Surgical Specialist in Salonica. After a period of postgraduate study in London he was appointed Surgeon to the Frodingham Hospital, and in 1926 Surgeon to the Northampton General Hospital. He was a keen supporter of the British Medical Association and Secretary of the Northamptonshire Branch. His interests were at all times strongly gynæcological; he was Obstetric

Consultant to several Borough and County Councils, and was keenly interested in the new Maternity Block at the Northampton General Hospital, to the control of which it was generally anticipated he would have been appointed but for his untimely death. He leaves a widow and two daughters, to whom we extend our sincere sympathy.

